# Proton Therapy for Head and Neck Adenoid Cystic Carcinoma: A Multi-institutional Review

**DOI:** 10.1016/j.ijpt.2026.101320

**Published:** 2026-05-10

**Authors:** Lisa A. McGee, Astha Rohit, Matthew Buras, Gopal Bajaj, John H. Chang, Noah S. Kalman, Ryan Grover, Adil Akthar, Adam J. Kole, Mark McDonald, Sanford Katz, Nancy Lee, Henry Tsai, James Urbanic, Carlos Vargas, Jing Zeng, Jason Molitoris, Scott Lester, Homan Mohammadi, Adam Holtzman

**Affiliations:** 1 Department of Radiation Oncology, Mayo Clinic, Phoenix, AZ, USA; 2 College of Medicine, Drexel University, Philadelphia, PA, USA; 3 Department of Quantitative Health Sciences, Division of Clinical Trials and Biostatistics, Mayo Clinic, Phoenix, AZ, USA; 4 Radiation Oncology Associates, PC, Fairfax, VA, USA; 5 Department of Radiation Oncology, Oklahoma Proton Center, Oklahoma City, OK, USA; 6 Department of Oncological Sciences, Herbert Wertheim College of Medicine, Florida International University and Miami Cancer Institute, Baptist Health South Florida, Miami, FL, USA; 7 Department of Radiation Oncology, Covenant Health, Knoxville, TN, USA; 8 Department of Radiation Oncology, Northwestern Medicine Proton Center, Warrenville, IL, USA; 9 Department of Radiation Oncology, University of Alabama, Birmingham, AL, USA; 10 Department of Radiation Oncology, Emory Proton Therapy Center, Atlanta, GA, USA; 11 Department of Radiation Oncology, Willis-Knighton Cancer Center, Shreveport, LA, USA; 12 Department of Radiation Oncology, Memorial Sloan Kettering Cancer Center, New York, NY, USA; 13 Department of Radiation Oncology, ProCure Proton Therapy Center, Somerset, NJ, USA; 14 Department of Radiation Oncology, University of California San Diego, San Diego, CA, USA; 15 Department of Radiation Oncology, Fred Hutch Cancer Center, Seattle, WA, USA; 16 Department of Radiation Oncology, Maryland Proton Treatment Center, Baltimore, MD, USA; 17 Department of Radiation Oncology, Mayo Clinic, Rochester, MN, USA; 18 Department of Radiation Oncology, Mayo Clinic, Jacksonville, FL, USA

**Keywords:** Proton beam therapy, Adenoid cystic carcinoma, Salivary gland cancer

## Abstract

**Purpose:**

To evaluate efficacy and toxicity (CTCAE v4.0) outcomes in patients with adenoid cystic carcinoma (ACC) treated with proton beam therapy (PBT).

**Patients and Methods:**

From 2012 through 2023, 79 patients with non-metastatic ACC were treated with PBT and enrolled on the Proton Collaborative Group (PCG) registry. Kaplan-Meier analyses quantified locoregional control (LRC), disease-free survival (DFS), and overall survival (OS). ACC patients receiving reirradiation with PBT were excluded from this analysis.

**Results:**

Median follow-up was 3 years (0.01–6.72). Twenty-six patients were unable to undergo surgery and received definitive PBT versus postoperative PBT (n=53). Median postoperative PBT dose was 66 GyE and 70 GyE for definitive patients treated with conventional fractionation. Most patients had localized disease (n=75); few had nodal metastases (n=4). 62% were locally advanced (T3-T4) at the time of PBT treatment. 3-year Locoregional control (LRC) for all patients was 98% [95% CI (94.3, 100)]. Advanced T-staging, positive margins and definitive-intent PBT were not associated with worsened LRC. 3-year Progression free survival (PFS) was 80.5% (70.6, 91.7). Patients receiving definitive intent PBT had lower but nonsignificant PFS (73.3% vs. 83.8%, p-0.075). Advanced T-staging and positive margins did not have worsened PFS. Overall survival at 3 years was 89.2% (81.4, 97.8). Acute grade 3 toxicities occurred in 16 patients, the most common included the following: mucositis (n=5), oral pain (n=3), and dermatitis (n=5). Late grade 3 toxicity (n=2) included middle ear inflammation (n=1) and skin ulceration (n=1). There were no grade 4+ toxicities.

**Conclusion:**

PBT appears to be highly efficacious in providing LRC for patients with ACC including those patients treated with definitive intent. PBT toxicity was acceptable. Longer follow-up is needed.

## Introduction

Adenoid cystic carcinomas (ACCs) are rare relatively slow-growing malignancies that arise most commonly from salivary glands in the head and neck.^1^, ^2^, ^3^, ^4^, ^5^, ^6^, ^7^, ^8^, ^9^, ^10^, ^11^ Hallmark features of this malignancy include its tendency for neurotropic spread resulting in perineural invasion (PNI), and eventual hematogenous spread most commonly resulting in pulmonary metastases.^2^, ^3^, ^4^, ^9^, ^10^, ^11^, ^12^ Regional spread to lymph nodes is rare.^2^, ^3^, ^4^, ^9^, ^10^, ^11^, ^12^

ACCs account for 1% of all head and neck cancers and represent 10% of all major salivary gland cancers and 30% of minor salivary gland cancers.^1^, ^3^, ^4^, ^9^, ^11^ Due the rarity of this disease and unique anatomical and staging characteristics of each patient, there is little prospective published data to guide treatment recommendations. Treatment paradigms are therefore based off of retrospective reviews and phase II data; no phase III data have been reported.^9^, ^11^, ^12^, ^13^ Historically, the published data grouped ACC treatment outcomes with other histological subtypes of salivary gland cancers without regard to the unique characteristics of ACC. The commonly accepted treatment paradigm is resection to negative margins if possible followed by adjuvant radiotherapy (RT) versus RT if the patient is unresectable.^14^ Local control is improved for those patients able to undergo surgical excision compared to definitive RT alone.^6^, ^15^, ^16^ Optimizing local control and cure with aggressive surgery and RT must be balanced against long-term toxicities which negatively impact patient quality of life with exacerbated complications if tumor PNI necessitates high dose treatment to the skull base.^7^, ^17^, ^18^

Historically, X-ray-based RT has been the mainstay of RT treatment of ACCs. However, these historical series have reported suboptimal local control at 5 years ranging from 26% to 61% which have supported the notion that ACCs are radioresistant.^5^, ^6^, ^8^, ^15^, ^16^, ^19^, ^20^, ^21^, ^22^, ^23^, ^24^ It has been suggested that higher linear energy transfer (LET) modalities such as particle therapy may help to overcome radioresistance.^25^, ^26^, ^27^, ^28^ A randomized trial of patients with salivary gland cancers demonstrated improvement in 10-year local control in those patients treated with fast neutron therapy (FNT) (56%) versus XRT (17%) although FNT resulted in higher rates of toxicity.^23^ This concept was later confirmed with retrospective data of ACC patients treated with FNT and XRT resulting in a 5-year local control of 75% versus 32%, with FNT again causing higher rates of toxicity.^5^

In recent years, construction of cancer centers with alternate forms of particle therapy such as proton beam therapy (PBT) have increased which make this modality of radiation more accessible to larger patient groups.^29^ It is hypothesized that other forms of particle therapy should also result in a high level of cure as evidenced by FNT data.^5^, ^23^ Additionally, the physical properties of PBT may allow for better dose sparing of adjacent organs which would mitigate concern for toxicity which persists from the FNT data.^6^, ^26^, ^27^, ^30^ This study reports treatment outcomes of patients with ACC treated with PBT on a multi-institutional prospective registry.

## Patients and methods

The Proton Collaborative Group (PCG) is a research consortium composed of twenty-four institutions in the United States and maintains a prospective registry of patients treated with PBT. The existing database was queried for all enrolled patients with ACC of the head and neck treated with PBT from 2012 to 2023. Patients were included if they received either definitive or adjuvant PBT. Patients receiving PBT reirradiation or who had distant metastatic disease at initial presentation were excluded from this series. Thirteen PCG sites contributed data to this series. Prior to treatment, all patients had ACC cancer stage assigned by the treating physician based on physical exam, diagnostic imaging including diagnostic CT and PET-CT per American Joint Committee on Cancer guidelines. MRI was used to assess for PNI when indicated in symptomatic or advanced stage patients. Patients were treated according to National Comprehensive Cancer Network (NCCN) guidelines per the treating radiation oncologist. Following treatment completion, follow-up was performed according to NCCN guidelines which included history and physical exam every 3 months for the first two years post treatment and every 6 months for years 3–5 post-treatment. Post-treatment PET-CT was performed 3 months post-treatment and then at the discretion of the treating physician. After each follow-up visit, data was submitted electronically to PCG and entered into the PCG database. De-identified patient demographics, treatment-specific details, and toxicity information were obtained from the PCG database for this report.

Locoregional control (LRC), progression-free survival (PFS) and overall survival (OS) were reported from date of PBT initiation to the event of interest and censored at the time of last follow-up. Kaplan-Meier (KM) analyses were used to estimate survival probabilities at 3 years with 95% confidence intervals (CI), with 100% survival having a 95% CI of (100, 100). The logrank test was used to assess the separation of the KM curves between groups. Numeric variables were summarized as median and interquartile range (IQR) as (Q1, Q3) while categorical variables were summarized as count (percent). Acute adverse events were defined as those that occurred during or within 180 days of PBT initiation. Late adverse effects were defined as those occurring > 180 days after the start of PBT. Hypothesis tests were two-sided with p<0.05 considered statistically significant. All analyses were conducted in R v4.4.1.^31^

## Results

Between 2012 and 2023, a total of 79 patients received PBT for nonmetastatic ACC of the head and neck. Patient, tumor, and treatment characteristics are reported in Table 1 and stratified according to definitive versus postoperative PBT. 48.1% (n=38) were female and 51.9% (n=41) were male. Median age of the cohort was 57 years. 30 patients developed ACC in a major salivary gland, parotid (n=17), submandibular gland (n=12), or sublingual gland (n=1). 46 patients developed ACC in a minor salivary gland, and three patients did not have their primary site within the head and neck reported. The majority of patients had T3/T4 disease (n=49) versus T1-T2 (n=30). Four patients (5%) had lymph node positive disease. Histologic grade of tumor was not reported in the majority of patients (n=59). The majority of patients had up-front surgery (n=53) while 26 patients received definitive PBT alone. Of those patients who had surgery, eight patients had negative margins, 20 patients had positive margins, and 25 patients did not have margin status reported. For those patients receiving postoperative RT, median PBT dose was 66 GyE (IQR (58.8, 70)). For patients receiving definitive-intent PBT, median PBT dose for those patients treated with conventional fractionation was 70 GyE (IQR (66, 72)). Three patients receiving definitive PBT were treated with a hypofractionated approach. Ten patients received concurrent chemotherapy during their PBT treatment, four of which received definitive PBT. Nine patients received concurrent cisplatin and one patient received concurrent carboplatin and paclitaxel.

**Table 1:** Patient and Treatment Characteristics.

Patient and treatment characteristics	Definitive PBT patients (n=26)	Postoperative PBT patients (n=53)
Gender		
Male Female	13 (16.5%) 13 (16.5%)	28 (35.4%) 25 (31.6%)
Histologic Grade		
Grade 1 Grade 2 Grade 3 Not reported	0 (0%) 4 (5.1%) 1 (1.3%) 21 (26.6%)	3 (3.8%) 6 (7.6%) 6 (7.6%) 38 (48.0%)
ECOG Performance Status		
0 1 3 Not Reported	13 (16.5%) 6 (7.6%) 1 (1.3%) 6 (7.6%)	32 (40.5%) 11 (13.8%) 0 (0%) 10 (12.7%)
Primary Site		
Major Salivary Gland Parotid Submandibular Gland Sublingual Gland Minor Salivary Gland Oral Cavity Oropharynx Nasopharynx Paranasal Sinuses Not reported	6 (7.6%) 3 (3.8%) 3 (3.8%) 0 (0%) 19 (24.1%) 0 (0%) 4 (5.1%) 6 (7.6%) 9 (11.4%) 1 (1.3%)	24 (30.4%) 14 (17.7%) 9 (11.4%) 1(1.3%) 27 (34.2%) 13 (16.5%) 1 (1.3%) 3 (3.8%) 10 (12.7%) 2 (2.5%)
T-Stage		
T1 T2 T3 T4	1 (1.3%) 3 (3.8%) 2 (2.5%) 20 (25.3%)	16 (20.3%) 10 (12.7%) 8 (10.1%) 19 (24.0%)
N-Stage		
N0 N1 N2 N3	25 (31.6%) 1 (1.3%) 0 (0%) 0 (0%)	50 (63.3%) 2 (25.3%) 1 (1.3%) 0 (0%)
Overall Stage		
I II III IV	1 (1.3%) 3 (3.8%) 2 (2.5%) 20 (25.3%)	16 (20.3%) 10(12.7%) 7 (8.9%) 20 (25.3%)
Margin Status		
Negative Margins Positive Margins Margins Not Reported	Not Applicable Not Applicable Not Applicable	8 (10.1%) 20 (25.3%) 25 (31.6%)
PBT Intent	26 (32.9%)	53 (67.1%)
Concurrent Chemotherapy	4 (5.1%)	6 (8.0%)
Treatment Center		
Fred Hutch Cancer Center Northwestern Medicine Proton Center Procure New Jersey Maryland Proton Treatment Center Inova Proton Therapy Center California Proton Therapy Center Oklahoma Proton Center Mayo Clinic Arizona New York Proton Center Miami Cancer Institute University of Alabama Proton Center Corewell Health Proton Therapy Willis-Knighton Health	13 (16.5%) 4 (5.1%) 2 (2.5%) 0 (0%) 2 (2.5%) 1 (1.3%) 1 (1.3%) 0 (0%) 0 (0%) 1 (1.3%) 0 (0%) 1 (1.3%) 1 (1.3%)	6 (7.6%) 13 (16.5%) 13 (16.5%) 6 (7.6%) 3 (3.8%) 3 (3.8%) 3 (3.8%) 2 (2.5%) 2 (2.5%) 1 (1.3%) 1 (1.3%) 0 (0%) 0 (0%)

**Figure 1:**
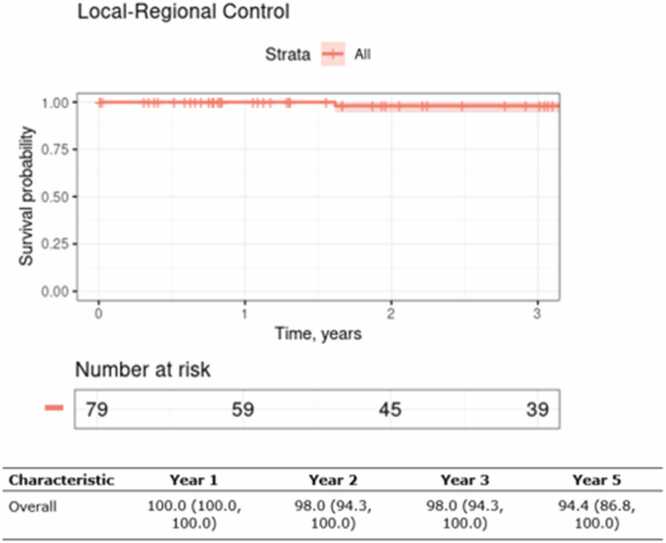
Locoregional control for the overall cohort.

Median follow-up was 3 years (Range; 0.01–6.7 years). Three-year LRC for the entire cohort was 98% [95% CI (94.3, 100)] (Figure 1). Three-year LRC for unresectable patients receiving definitive PBT was 94.1% [95% CI (83.6, 100]. T-staging, margin status, and PBT intent were not statistically significantly associated with LRC. Two patients, both with T4 N0 paranasal sinus ACC with gross perineural invasion to the skull base experienced local recurrence following treatment with definitive PBT without concurrent chemotherapy.

**Figure 2:**
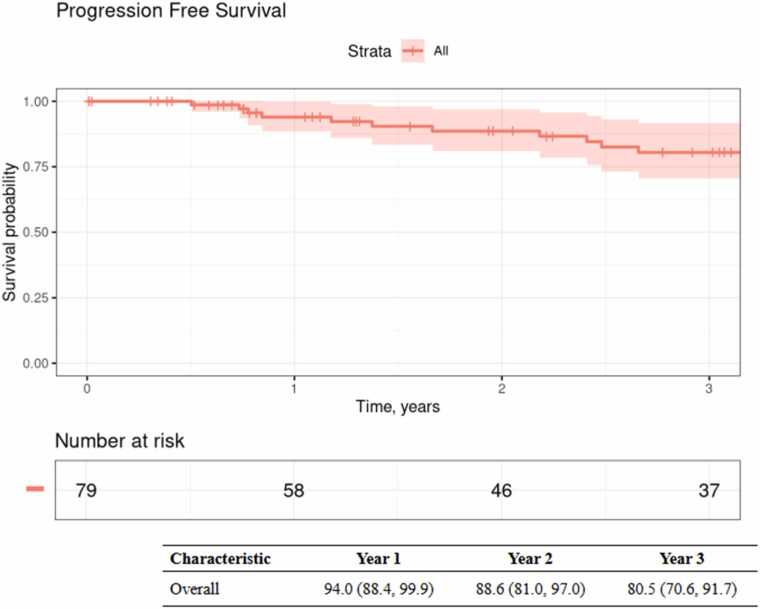
Progression-free survival for the overall cohort.

**Figure 3:**
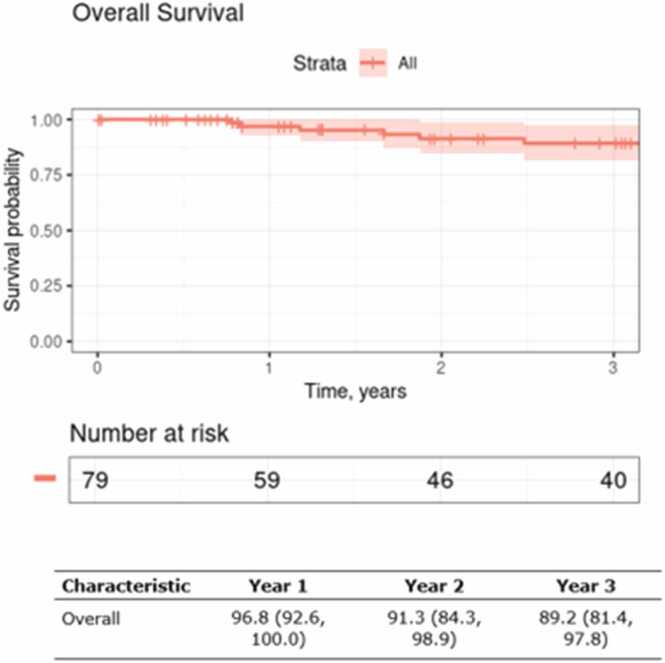
Overall survival for the overall cohort.

Three-year PFS was 80.5% (70.6, 91.7) (Figure 2). Patients treated with definitive intent PBT had lower but nonsignificant 3-year PFS compared to patients who received surgery plus adjuvant PBT (73.3% (55.5, 96.9) vs. 83.8% (72.7, 96.7), P=0.075). Three-year OS of the entire cohort was 89.2% (81.4, 97.8) (Figure 3). Patients with advanced T staging (T3-T4) had inferior three-year OS (83.3% (71.8, 96.5) vs 100%, p=0.043), whereas margin status and PBT intent did not statistically significantly impact OS.

In total, sixteen patients experienced acute grade 3 toxicity, the most common being dermatitis, mucositis, and oral pain (Table 2). Two patients experienced late grade 3 toxicity. One patient with T4 N0 ACC of the left parotid treated with subtotal resection and postoperative PBT to 70 GyE experienced late grade 3 middle ear inflammation. Another patient with T4 N0 ACC of the maxillary sinus treated with subtotal resection and postoperative PBT to 70 GyE experienced late grade 3 skin ulceration. There were no grade 4+ toxicities.

**Table 2:** Grade 3 Toxicity

Grade 3 Toxicity	Time	Patients (N)
Anorexia	Acute	1
Dermatitis	Acute	5
Dysphagia	Acute	1
Esophagitis	Acute	1
Oral Pain	Acute	3
Oral Mucositis	Acute	5
Nausea	Acute	1
Middle Ear Inflammation	Late	1
Skin Ulceration	Late	1

## Discussion

ACC is a rare salivary gland malignancy characterized by indolent growth and a high propensity for PNI.^1^, ^2^, ^3^, ^4^, ^5^, ^6^, ^7^, ^8^, ^9^, ^10^, ^17^, ^25^ The current standard treatment paradigm for resectable disease consists of surgical resection to negative margins followed by postoperative RT, while definitive-intent high-dose RT is recommended for patients with inoperable disease.^14^, ^32^, ^33^, ^34^ Tumor extension along major cranial nerves often complicates management, as skull base invasion may render patients unresectable and necessitate delivery of high dose RT in close proximity to critical organs at risk which can limit desired target coverage with prescription dose. Historical data from X-ray-based RT have demonstrated that postoperative RT improves local control for high grade histology, and locally advanced disease (stage III/IV) including those patients with positive lymph nodes.^32^, ^33^ A separate series demonstrated higher rates of local failure in patients who omitted postoperative RT, had advanced stage (stage III/IV), close or positive margins or PNI.^34^ Based on these data, commonly accepted indications for postoperative RT include advanced stage (T3-T4), intermediate or high-grade histology, PNI, lymphovascular space invasion, close or positive margins or positive lymph nodes. Despite this, historical treatment outcome series utilizing X-ray-based techniques have reported suboptimal 5-year LC/LRC between 26% and 61%.^5^, ^15^, ^16^, ^19^, ^20^, ^21^, ^22^, ^23^, ^24^

Particle therapy with higher LET and higher relative biological effectiveness (RBE) may be able to overcome the relative radioresistance of ACC.^6^, ^11^, ^18^, ^25^, ^26^, ^27^, ^28^, ^30^, ^35^ However, the optimal RBE, and therefore the optimal particle modality, for the treatment of ACC remains uncertain. In PBT, a standard RBE of 1.1 is universally applied in clinical treatment planning.^36^ Nonetheless, RBE in PBT is not constant and may increase, approaching values near 2, depending on factors such as cell type, dose per fraction, and position along the Bragg peak.^36^ Despite this variability, the effective RBE of protons generally remains lower than the biologically estimated RBE range of approximately 2–3 reported for carbon ion radiotherapy (CIRT) and 2–4 for fast neutron therapy (FNT).^37^, ^38^

Data from FNT have confirmed superior efficacy to X-ray-based RT for treatment of salivary gland cancers at the cost of worsened toxicity.^17^, ^19^, ^25^, ^39^, ^40^ Therefore, alternative forms of particle therapy such as PBT and CIRT warrant further investigation. In addition to having a slightly higher RBE than x-ray-based RT, the physical properties of PBT allow for improved conformality of treatment plans and may allow for improved target coverage while simultaneously sparing adjacent organs at risk.^6^, ^13^, ^20^, ^27^, ^30^, ^41^, ^42^, ^43^, ^44^, ^45^, ^46^, ^47^, ^48^, ^49^, ^50^ These dosimetric characteristics could result in improved efficacy compared to X-ray-based techniques while limiting the toxicities observed with FNT. A review of the literature evaluating particle therapy for ACC has demonstrated higher rates of local control with PBT compared to CIRT and FNT.^13^ The reasons for this observation remain unclear and may reflect factors beyond intrinsic RBE differences. It is therefore possible that the RBE achieved with PBT is sufficient for effective tumor control in ACC. Importantly, cross-study comparisons from this literature review are limited by the retrospective nature of available data, including potential selection bias, differences in treatment accessibility, variability in follow-up duration, and heterogeneity in patient populations. Furthermore, the success of particle therapy in ACC is multifactorial and depends not only on RBE, but also on the ability to safely escalate dose, achieve superior conformality, particularly at the skull base where PNI is common, and optimize fractionation strategies.

This multi-institutional retrospective series pools data from thirteen PCG member sites and represents one of the larger ACC series treated with PBT reported to date. Our findings support the hypothesis that particle therapy such as PBT is associated with improved disease control, with a 3-year LRC rate of 98%, exceeding historical outcomes reported with X-ray-based RT. Importantly, LRC remained favorable even among patients with locally advanced disease (T3–T4) and those treated with definitive-intent PBT due to unresectability. Given the small number of locoregional failures, it is not unexpected that advanced T stage, nodal involvement, close or positive margins, and definitive-intent treatment were not associated with inferior LRC in this cohort, in contrast to findings reported in historical photon-based series. Three-year PFS for the entire cohort was 80.5%. Patients treated with definitive-intent PBT experienced worse although nonsignificant PFS compared with those treated in the adjuvant setting, reflecting a higher burden of advanced disease and therefore a greater risk of distant metastases in the definitive cohort. Given the limited number of progression events, no statistically significant decrements in PFS were observed among patients with advanced T stage or close or positive margins. OS was also favorable.

This series adds to the current body of literature supporting the efficacy PBT has for patients with ACC. In total, there are eighteen retrospective studies, ranging from 5 to 129 patients, from 2009 to 2025, 13 of which report on ACC histology alone and four that report mixed histology series treated with PBT.^6^, ^13^, ^20^, ^27^, ^30^, ^41^, ^42^, ^43^, ^44^, ^45^, ^46^, ^47^, ^48^, ^49^, ^50^ A literature review has demonstrated that ACC patients in these series were more likely to present with localized disease, although T-staging was more likely to be advanced, T3-T4 (median 92%, range 33–100%).^13^ Throughout these series, a median of 73% of patients underwent up-front surgery.^13^ These series also report high rates of 2-year LC (86–100%) in patients treated with PBT.^13^ 5-year OS ranged from 59% to 89%.^13^

This series also confirms that PBT is associated with acceptable toxicity profiles, comparable to historical X-ray-based controls. Sixteen patients experienced transient grade 3 acute toxicities during treatment. Only two patients experienced late grade 3 toxicities, and no grade 4 or higher events were observed. Reported grade ≥3 toxicities in other series have been uncommon and typically include acute mucositis or dermatitis and, less frequently, late visual impairment.^13^

These data have limitations including selection bias of the patients who were treated with PBT. Many PBT centers in the United States are at academic centers and often in larger communities so not all patients in a catchment area may have equitable access to PBT care. There is also bias potential in treatment approach based on individual institutional preferences, such as how aggressive the surgical team is at obtaining clear margins. Additionally, there is a bias of confounding indication, meaning that patients who are selected to receive PBT are those patients the treating physicians may expect to benefit most. Longer follow-up is needed to assess longitudinal treatment outcome trends. This data represents a patient population with a rare malignancy treated with PBT for which access may be limited in many geographical areas in the United States. Patients therefore often travel to the PBT center for treatment and seek follow-up care close to home which limits long-term follow-up data from being reported by the member institutions. Likewise, there may be under reporting of late toxicities since many of the patients are followed by their local healthcare team. Lastly, information collected may have misclassification bias since this data was collected without being targeted by a predefined research hypothesis. Examples of misclassification bias for this cohort were the lack of reporting of clinical versus pathological PNI, lack of reporting of histological ACC subtype, inconsistent reporting of tumor grade as well as lack of reporting whether or not elective nodal coverage was pursued, all of which would be variables likely to correlate to treatment outcomes.

## Conclusion

This series from the PCG demonstrated excellent efficacy for PBT treatment for ACC of the head and neck, even in those patients who were unresectable and treated with definitive intent. PBT-associated toxicity was acceptable. Longer follow-up is needed to evaluate the durability of treatment outcomes.

## Conflicts of Interest

Dr. Molitoris does consulting work with Varian and Semens which is unrelated to proton therapy. The rest of the authors do not have any disclosures.

## Data Sharing Statement

Data may be made available upon reasonable request.

## CRediT authorship contribution statement

Lisa McGee: Conceptualization, Data curation, Formal analysis, Investigation, Methodology, Project administration, Resources, Validation, Writing - original draft, Writing - review and editing. Astha Rohit: Conceptualization, Methodology, Writing - original draft, Writing - review and editing. Matthew Buras: Data curation, Formal analysis, Methodology, Validation, Writing - original draft, Writing - review and editing. Gopal Bajaj: Conceptualization, Investigation, Methodology, Writing - review and editing. John H Chang: Conceptualization, Investigation, Methodology, Writing - review and editing. Noah Kalman: Conceptualization, Investigation, Methodology, Writing - review and editing. Ryan Grover: Conceptualization, Investigation, Methodology, Writing - review and editing. Stephen Mihalcik: Conceptualization, Investigation, Methodology, Writing - review and editing. Adam Kole: Conceptualization, Investigation, Methodology, Writing - review and editing. Mark McDonald: Conceptualization, Investigation, Methodology, Writing - review and editing. Sanford Katz: Conceptualization, Investigation, Methodology, Writing - review and editing. Nancy Lee: Conceptualization, Investigation, Methodology, Writing - review and editing. Henry Tsai: Conceptualization, Investigation, Methodology, Writing - review and editing. James Urbanic: Conceptualization, Investigation, Methodology, Writing - review and editing. Carlos Vargas: Conceptualization, Investigation, Methodology, Writing - review and editing. Jing Zeng: Conceptualization, Investigation, Methodology, Writing - review and editing. Jason Molitoris: Conceptualization, Investigation, Methodology, Writing - review and editing. Scott Lester: Conceptualization, Investigation, Methodology, Writing - review and editing. Homan Mahammadi: Conceptualization, Investigation, Methodology, Writing - review and editing. Adam Holtzman: Conceptualization, Investigation, Methodology, Writing - review and editing.

## Declaration of Competing Interest

The authors declare the following financial interests/personal relationships which may be considered as potential competing interests: Jason Molitoris reports a relationship with Varian Medical Systems Inc that includes: consulting or advisory. Jason Molitoris, MD reports a relationship with Siemens AG that includes: consulting or advisory. If there are other authors, they declare that they have no known competing financial interests or personal relationships that could have appeared to influence the work reported in this paper.
